# Evaluation of Cross-Species Transferability of SSR Markers in *Foeniculum vulgare*

**DOI:** 10.3390/plants9020175

**Published:** 2020-02-01

**Authors:** Domenico Aiello, Nicoletta Ferradini, Lorenzo Torelli, Chiara Volpi, Joep Lambalk, Luigi Russi, Emidio Albertini

**Affiliations:** 1 Department of Agricultural, Food and Environmental Sciences, University of Perugia, 06121 Perugia, Italy; domenico.aiello@studenti.unipg.it (D.A.); nicoletta.ferradini@gmail.com (N.F.); lorenzo.torelli95@libero.it (L.T.); luigi.russi@unipg.it (L.R.); 2 Enza Zaden Italia Research S.r.l. SS., 01016 Tarquinia, Italy; C.Volpi@enzazaden.it; 3 Enza Zaden, Research and Development B.V. P.O. Box 7, 1600AA Enkhuizen, The Netherlands; J.lambalk@enzazaden.nl

**Keywords:** *Foeniculum vulgare*, fennel, *Daucus carota*, SSR, cross-species

## Abstract

Fennel (*Foeniculum vulgare*) is a species belonging to the Apiaceae family, well known for its nutritional and pharmacological properties. Despite the economic and agricultural relevance, its genomic and transcriptomic data remain poor. Microsatellites—also known as simple sequence repeats (SSRs)—are codominant markers widely used to perform cross-amplification tests starting from markers developed in related species. SSRs represent a powerful tool, especially for those species lacking genomic information. In this study, a set of primers previously designed in *Daucus carota* for polymorphic SSR loci was tested in commercial varieties and breeding lines of fennel in order to: (i) test their cross-genera transferability, (ii) look at their efficiency in assessing genetic diversity, and (iii) identify their usefulness for marker-assisted selection (MAS) in breeding programs. Thirty-nine SSR markers from carrot were selected and tested for their transferability score, and only 23% of them resulted suitable for fennel. The low rate of SSR transferability between the two species evidences the difficulties of the use of genomic SSR in cross-genera transferability.

## 1. Introduction

*Foeniculum vulgare* Mill. (2n = 22), commonly known as fennel, is a cross-pollinating, herbaceous plant belonging to the Apiaceae family (Umbelliferaceae). It is native of the southern Mediterranean regions and nowadays, through naturalization and cultivation, grows wild also in Asia, North America, and Europe [1,2,3]. It is a hardy umbelliferous annual, biennial, or perennial aromatic herb and comprises two subspecies: *F. vulgare* ssp. *piperitum* (Ucria) Countinho and *F. vulgare* ssp. *capillaceum* (Gilib.) Holomboe. While the first grows only wild and is characterized by its very bitter fruits, the latter is the cultivated form, used mainly as food, and characterized by fruits with a lower level of bitterness. *F. vulgare* ssp. *capillaceum* includes three botanical varieties: var. *vulgare* (Mill) Thell., var. *dulce* (Mill) Thell., with a sweet taste due to the low fenchone content in the essential oil, and var. *azoricum* (Mill) Thell., known as Italian fennel and probably stemming by one of the above varieties by selection [3,4,5]. The first two varieties are used as flavor agents in several fresh and packaged products, while the third is cultivated as a vegetable [6].

*F. vulgare* is well known and utilized since antiquity for its taste and medical properties [3,7,8,9], and also as a magic and religious remedy [10,11,12]. While the ancient Egyptians and Greeks used it as food and medicine, in China it was considered a snake bite remedy, and Romans and Indians grew it for its aromatic fruits [3,13,14,15]. Fennel is used throughout the world in folk medicine for the treatment of many common diseases. Nowadays, its herbal remedial and essential oils are widely used for abdominal pains, arthritis, colics in children, conjunctivitis, constipation, diarrhea, fever, flatulence, gastritis, insomnia, irritable colon, liver pain, mouth ulcer, stomachache, and other conditions [2,3,16,17,18]. Moreover, several studies have reported not only its antioxidant, anti-cancer, anti-microbial, and anti-fungal properties, but also its hepatoprotective, hypoglycemic, and estrogenic activities [3,18,19]. Fennel contains various minerals and trace elements [20], fat- and water-soluble vitamins, amino acids, and essential oils [21,22,23].

Despite its interesting pharmaceutical properties and agronomic traits, fennel remains a genetically under-studied species. In fact, given the lack of genomic information available in the literature and in gene banks, dominant molecular markers for this species have been widely used to assess the genetic diversity of germplasm accessions and to investigate the genetic stability and uniformity of plants regenerated through organogenesis and embryogenesis [24,25,26,27]. These tools are widely applicable because they provide rapid results and do not require a prior design of primer sequences [28]. More recently, Maghsoudi Kelardashti et al. [29] used related amplified polymorphism (SRAP) to detect the genetic diversity in 11 fennel populations, obtaining a higher rate of polymorphisms than those reported by other authors using random amplified polymorphic DNA (RAPD), inter-simple sequence repeats (ISSRs), and amplified fragment length polymorphism (AFLPs). 

The development of more informative molecular markers, such as the simple sequence repeats (SSRs), and the use of advanced statistical tools could be of great aid in assessing the genetic variability within and between populations. SSRs are neutral markers, widely and successfully used to evaluate the genetic structure in different species [30]. Some advantages of using SSR markers are their locus specificity, highly reproducibility, and codominant nature. Furthermore, the high rate of polymorphisms and their large distribution throughout the genome made them the most used markers for breeding programs in plants [31,32,33,34]. The set-up of species-specific microsatellite markers is time-consuming and expensive and involves the development of enriched SSR libraries, the sequencing of the targeted genomic regions, and the design of flanking primers [35]. This is one of the reasons that has limited the development and use of SSR markers in species of scarce economic interest. Alternatively, a widely used strategy implements species-specific microsatellites through cross-species amplification based on species high closeness, without additional costs [36,37,38,39]. If this is the case, the best results are obtained by amplifying regions of species belonging to the same genus or to closely related genera. This means that the success in cross-amplification of any DNA sequence is inversely related to the evolutionary distance between two species [40]. 

Recently, Palumbo et al. [41] performed the fennel leaf transcriptome sequencing and identified several genes related to the biosynthesis of *t*-anethole, a compound well known for its nutraceutical and medical properties. Moreover, by screening the assembled transcriptome in the tested samples, they identified approximately 43,000 single-nucleotide polymorphisms (SNPs), 4000 indels and 6411 microsatellite regions. Of the latter, as many as 27 SSR markers were suitable for genetic diversity analyses [42].

In the present study, we used a set of polymorphic microsatellites, specifically designed in *Daucus carota* by Cavagnaro et al. [43], in order to: (i) test their cross-genera transferability, (ii) look at their efficiency in assessing genetic diversity, and (iii) identify their usefulness for marker-assisted selection (MAS) in breeding programs.

## 2. Results

**Figure 1:**
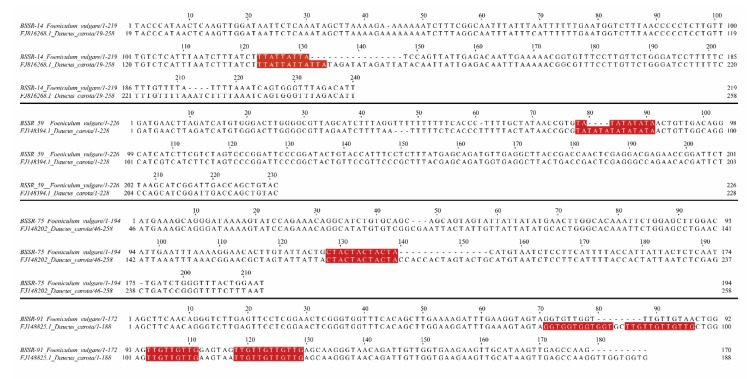
Sequence alignments between simple sequence repeat (SSR) flanking regions of carrot (from Cavagnaro et al. [43]) and fennel. Common SSR motifs are highlighted in red.

Out of 39 *D. carota* SSR markers tested for their transferability in fennel, 9 (23%) displayed clear and reliable amplicons of the expected size, and 7 showed a multiband profile indicating a general low rate of transferability. However, the sequence of all fennel amplicons generated by the 16 SSR primer pairs and the resulting alignment with those of the original carrots showed the presence of repetitive motifs in nine of them. This confirmed the observed low rate of SSR transferability between the two species. Moreover, a general low degree of conservation of the SSR flanking regions and a high mutation rate were observed. Only four loci (BSSR-14, BSSR-59, BSSR-75, and BSSR-91, see Figure 1) revealed a high percentage of similarity in the SSR flanking regions. Moreover, only six out of nine sequenced loci revealed the same repeat motifs in carrot and fennel, whereas the other three showed different repetitive motifs (Table 1 and Table 2).

The size of the SSRs varied from 6 bp to 22 bp for dinucleotide motifs (3 to 11 repeats) and from 9 to 12 bp for trinucleotide motifs (3 to 4 repeats). A new pair of primers was designed on the basis of the flanking regions of the repetitive motifs for SSRs that originally produced multiband profiles (GSSR-16, GSSR-35, and GSSR-138 – Table 2).

Scorable amplicons were produced for all nine nuclear SSRs, with a total of 30 alleles. Out of nine markers tested, three of them (GSSR-138, BSSR-14, BSSR-91) resulted monomorphic. The average number of alleles per locus was 3.33, ranging from one (GSSR-138, BSSR-14, and BSSR-91) to 12 (GSSR-91), but the number of effective alleles per locus was significantly lower (Na = 2.13). At locus BSSR-75, the allele 242 showed the highest frequency (0.95) (Table 3).

No rare alleles (frequency < 0.01, [44]) were found, whereas several private alleles were observed. Seven out of eight alleles at locus GSSR-154 were private, while one private allele was observed in the GSSR-97, GSSR-35, GSSR-16, and BSSR-75 loci.

The mean observed heterozygosity (Ho) was 0.172, ranging from 0.056 (locus GSSR-16) to 0.65 (GSSR-97) (Table 3). The mean expected heterozygosity (He) was 0.303, indicating a low variability between accessions. The highest values of heterozygosity were found at loci GSSR-154 and GSSR-97 (0.846 and 0.726, respectively), while the lowest value was found at locus BSSR-75 (0.097).

Of the nine analyzed loci, only two showed polymorphism information content (PIC) values ranging from 0.66 (GSSR-97) to 0.81 (GSSR-154) and were highly informative; all the others presented values lower than 0.5, indicating a general low allelic variation and, consequently, they were considered to be reasonably or slightly informative [45] (Table 1). Overall, out of nine investigated markers, six of them showed the presence of null alleles with estimated frequencies ranging from 0% to 80%. The highest values were observed for the markers GSSR-16 (0.8298) and BSSR-75 (0.7350), whereas marker GSSR-35 was the only one unaffected by null alleles.

**Figure 2:**
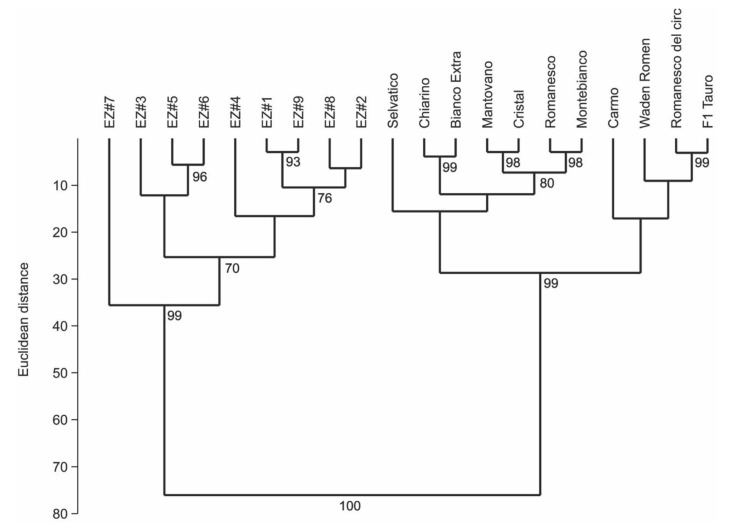
Ward’s clustering dendrogram of *F. vulgare* accessions.

Moreover, at a distance of 40 units, the SSR markers were able to clearly differentiate (*p* < 0.001) the fennel entries into two main groups (Figure 1): the first included all breeding lines provided by Enza Zaden, the second comprised a representative sample of old and recently bred fennel varieties. Interestingly, the germplasm of the first group is likely to come from unique sources, different from those of the second (Figure 2).

## 3. Discussion

Microsatellites are codominant markers characterized by high polymorphism and, because of this, are widely recognized as very powerful and informative in both animal and plant species [46]. This hypervariable nature of SSRs produces very high allelic variations, even among very closely related varieties. Therefore, they are considered the markers of choice for the characterization of core collections and for the management of germplasm collections. Moreover, one of the characteristics that makes these markers particularly interesting in genetic diversity studies is their high rate of transferability to closely related species [47,48,49,50,51]. Nevertheless, significantly low values of cross-transferability have been observed for genomic SSRs, which are known to be more polymorphic but located in less conserved regions of the genome [36,52]. In this regard, Liewlaksaneeyanawin et al. [53] compared the rate of transferability of SSRs developed from expressed sequence tags (ESTs), unscreened genomic DNA, low-copy genomic DNA, and undermethylated genomic DNA from Loblolly pine (*Pinus taeda*) to those obtained for other species, achieving a transferability success of 100, 29, 23, and 30%, respectively.

Due to the lack of specific markers for *F. vulgare,* we selected and tested 39 highly polymorphic *D. carota* specific genomic SSRs, already assayed by Cavagnaro et al. [43] for their cross-transferability in other Apiaceae species. Surprisingly, the rate of cross-genera transferability was very low (23%). This figure was significantly lower than the values of 41%, reported by Cavagnaro and collaborators [43], and 67%, reported by Cholin et al. [54] for the same markers, as well as than those found in the literature for other genera, reviewed by Rossetto [55]. In fact, average values of transferability across related genera, ranging between 10% and 71%, have been reported by several authors for *Cucumis, Vitis, Quercus, Helianthus*, and *Glycine* [56,57,58,59]. On the other hand, the success of cross-transferability depends upon the evolutionary distance between the source and the target species. The higher the genomic homology, the greater the conservation of SSR-flanking regions and, hence, the transferability of SSR markers [60]. Moreover, it is also worth considering that the degree of transferability and the level of polymorphism of a given SSR marker could be influenced by the level of ploidy, as well as by mutational events [61]. In our case, out of nine SSR loci, three (GSSR-16, GSSR-35, and GSSR-138) gave a multiband profile with fragments of the expected size of the original sequence. This phenomenon is common in SSRs due to multiple primer binding sites along the genome and to the amplification of homoeoloci [47,62]. The sequencing and alignment of these bands revealed a general low homology between the flanking regions and repetitive motifs of fennel and carrot (42.6%, 46.5%, and 37.4% identity, respectively). These finding are in contrast with the results obtained by Cholin et al. [54], who tested two of these loci (GSSR-16 and GSSR-35) in fennel and other Apiaceae species and observed a good amplification with a similar banding pattern to that of carrot. However, in our study, the fragments were not sequenced but only displayed on agarose gel.

While loci GSSR-4 and GSSR-111 amplified well in Cholin et al. [54] studies, we had to discard them, since no amplification was observed. This discrepancy could be explained by the fact that Colin et al. used a single entry per species so, eventually, they could not determine the presence of null alleles. Null alleles in microsatellite loci are the result of an insufficient PCR amplification due to a mutation(s) in the flanking sequence complementary to one of the oligonucleotide primers, resulting in a reduction of the observed heterozygosity and complicating the interpretation of the microsatellite data [63,64,65]. The association between the presence of null alleles and the highly variability of the flanking regions, due to their low stability with respect to other genomic regions, was extensively demonstrated [66,67]. These observations also support the hypothesis that the frequency of null allele increases rapidly with the phylogenetic distance among species [64]. For all these reasons, in a microsatellites cross-transferability study, sequencing is a mandatory step for understanding and correctly interpreting SSR data. In our study, the sequencing and alignment of fennel and carrot SSR loci revealed a general low degree of homology between the flanking regions and the repetitive motifs, and this could explain the observed low rates of transferability. Similar patterns of mutation, in terms of number of SSR repeat units, base substitution, and insertions/deletions (indels) within and outside the microsatellite motif were reported by other authors [68,69]. On the other hand, due to the lack of an EST–SSR bibliography in species closely related to fennel, we used genomic microsatellites known for their high polymorphism but low transferability, being slightly conserved.

Only four loci (BSSR-14, BSSR-59, BSSR-75, and BSSR-91) showed good homology of the flanking regions and repetitive motifs. This confirmed, once again, what has been already observed by other authors about the limited transferability of genomic microsatellites between species and between genera. In fact, although carrot’s SSR markers amplified successfully in fennel, the analysis of the flanking regions as well as the pattern and the number of the repetitions indicated that these regions are poorly conserved in these two species and, eventually, among Apiaceae taxa. This confirmed that indels and substitutions are more frequent among more distantly related species and represent the major mutational processes of gene evolution [70].

The number of alleles per locus detected in fennel ranged from 1 to 12, with a mean value of 3.3 alleles per locus, values much lower than those reported by Cavagnaro et al. [43] in carrot but higher than those reported by Cholin et al. [54] in fennel using carrot SSRs. Even if the level of polymorphism of the nine microsatellites used here resulted low (average PIC = 0.267) and only two markers (GSSR-97 and GSSR-154) were highly informative (PIC > 0.5), it was possible to clearly differentiate the fennel accessions into two main groups: one included all the breeding lines provided by Enza Zaden, and the other comprised a sample of old and new commercial varieties. This agrees with a positive correlation found between the length of the repetitive motifs and the level of polymorphism [46,71,72,73,74]. In fact, in this study, the two loci with the highest number of repetitions, GSSR-154 [(AC)_6_(AG)_9_] and GSSR-97 [(GA)_4_; (AG)_11_], were also the most polymorphic (PIC = 0.810 and 0.656, respectively). It is well known that an SSR mutation due to the expansion or contraction of a repeat’s length can occur due to replication slippage, errors during replication and repair, or recombination events [75,76,77].

In conclusion, our findings provide additional evidence of the difficulties of using genomic SSR in cross-genera transferability: it could be time-consuming, expensive, and not very effective in terms of transferability and level of polymorphisms. In addition, it is evident that, also for species of minor economic importance, EST data of closely related species should be exploited in order to identify informative SSR loci. Additionally, the use of more informative techniques such as the transcriptome sequencing analysis will provide useful molecular information for genetic and functional characterizations, as recently published for fennel [41].

## 4. Materials and Methods 

### 4.1. Plant Material and Genomic DNA Extraction

Eleven fennel commercial varieties and nine breeding lines (F_1_) provided by Enza Zaden (EZ, Tarquinia, Italy) company have been included in this study (Table 4). Total genomic DNA was isolated from young leaves using the GenElute Plant Genomic DNA Miniprep Kit (Sigma-Aldrich, St. Louis, Missouri, USA) according to the supplier’s specifications.

### 4.2. Primer Design and SSR Amplification by PCR

Thirty-nine genomic SSRs specific of *D. carota* [43] were used to evaluate cross-genera transferability. PCR reactions were performed in a total volume of 50 μL using 1X Phusion HF buffer, 200 μM of each dNTPs, 0.5 μM of each primer, 0.2 U of Phusion Taq DNA polymerase (Thermo Fischer Scientific, Waltham, Massachusetts, USA), and 20 ng of genomic DNA. All amplifications were carried out with a GeneAmp PCR system 9700 (Applied Biosystems, Foster City, California, USA) programmed as follow: 98 °C for 30 s, followed by 30 cycles of 98 °C for 10 s, 50–62 °C for 15 s, 72 °C for 30 s, and then 72 °C for 20 min.

The annealing temperature was lowered by 2–5 °C according to the evolutionary distance between species, as suggested by Rossetto [55]. PCR products were separated by 2% agarose gel electrophoresis: SSR markers which did not amplify in fennel were discarded, while those that showed one or two specific bands were selected, and the amplified fragments were ligated into the pCR4-TOPO TA Vector (Invitrogen – Thermo Fisher Scientific, Carlsbad, CA, USA). Three positive clones for each SSR marker were selected for sequencing on an ABI Prism 3130 sequencer (Applied Biosystems, Foster City, California, USA) using BigDye terminator V3.1 kit in a cycle sequencing protocol, according to the manufacturer’s specifications (Applied Biosystems, Foster City, CA, USA).

Vector’s sequences were removed, and the unique sequences were edited using the sequence assembly program (Vector NTI^®^ Express Software – Invitrogen TM, Carlsbad, CA, USA) and later screened for the presence of SSRs with the program Tandem Repeat Finder (Boston University, Boston, MA, USA) [78]. New fennel specific primers that flanked the microsatellites were designed using the Primer3 software (Whitehead Institute for Biomedical Research) [79] (Table 2).

PCRs were carried out with the Type-it Microsatellite PCR Kit (Qiagen, Hilden, Germany) containing 1X Type-it master mix with 0.2 μM of each fluorescent forward primer labelled with 6-FAM dyes (Sigma-Aldrich, St. Louis, Missouri, USA), and reverse unlabeled primer and 20 ng of DNA and H_2_O, to a final volume of 20 μL. Amplifications were performed as follow: an initial step at 95 °C for 5 min, followed by 30 cycles at 95 °C for 30 s, 50–62 °C for 30 s, and 72 °C for 30 s, and a final extension at 72°C for 10 min. All amplifications were performed in a GeneAmp PCR System 9700 (Applied Biosystems, Foster City, CA, USA).

PCR products were denaturated at 95 °C for 5 min, separated and analyzed using a 3130 XL DNA Analyzer (Applied Biosystems, Foster City, CA, USA). The size of the amplified products was determined with respect to an internal standard DNA (GeneScan 500 Liz, Thermo Fischer Scientific, Waltham, MA, USA), and the scorable peaks were assigned using GeneMapper software (Applied Biosystems, Foster City, CA, USA).

### 4.3. Data Analysis 

GenAlEx version 6.5 [80] program was used to measure the number of alleles (Na) per locus, the effective number of alleles (Ne), the percentage of rare alleles (RA = allele frequency < 0.01), the observed heterozygosity (Ho), the gene diversity/expected heterozygosity (He). The polymorphism information content (PIC) for each SSR was calculated with the program CERVUS version 2.0 (Field Genetic Ltd, London, UK), using the following formula:

$$\mathrm{PIC}=1-\overset{n}{\underset{i=1}{\sum }}p_{i}^{2}-\left(\overset{n}{\underset{i=n}{\sum }}p_{i}^{2}\right)+\overset{n}{\underset{i=n}{\sum }}p_{i}^{4}$$

The analysis also included the probability of identity (PID) [81] and the probability of identity among sibs PID/sib [82], calculated as follows:

$$P_{ID}=\sum^{}p_{i}^{4}+\sum^{}\sum^{}{\left(2p_{i}p_{j}\right)}^{2}$$

$$P\left(\mathrm{ID}\right)\mathrm{sib}=0.25+\left(0.5\sum^{}p_{i}^{2}\right)+\left[0.5{\left(\sum^{}p_{i}^{2}\right)}^{2}\right]-\left(0.25\sum^{}p_{i}^{4}\right)$$

Finally, the ability of each marker to discriminate two random cultivars was estimated by the power of discrimination (PD = 1-PID) [83].

The SSR data were used to compute a Euclidean distance matrix, and the 21 accessions were clustered by Ward’s hierarchical method [84] and validated by 1000 bootstrap replicates using PAST software (University of Oslo, Norway) [85].

## Figures and Tables

**Table 1:** SSR motifs comparison between *Daucus carota* from Cavagnaro et al. [43] and *Foeniculum vulgare*.

Locus	Accession No.	SSR Motifs	
		*D. carota*	*F. vulgare*
GSSR-16	Fj816126	(TG)_9_ tacgc (ATGT)_3_	(AT)_3_
GSSR-35	Fj816145	(GA)_13_	(GA)_9_
GSSR-97	Fj816206	(GA)_8_(AG)_7_ aagtattcca(AG)_6_(GA)_7_	(GA)_4_; (AG)_11_
GSSR-138	Fj816246	(GT)_5_ ata (GT)_7_(AG)_21_	(GA)_3_
GSSR-154	Fj816262	(TC)11	(TC)_5_ (TC)_13_
BSSR-14	FJ816268	(TTA)_4_	(TTA)_3_
BSSR-53	Fj148355	(AT)_8_	(TG)_3_ (TA)_4_
BSSR-59	Fj148394	(TA)_7_	(TA)5
BSSR-75	Fj148202	(TAC)_5_	(GCA)_3_; (TAT)_3_; (CTA)_4_
BSSR-91	Fj148825	(GGT)_4_ gc(TTG)_4_(TTG)_4_	(TTG)_3_(TTG)_4_

**Table 2:** SSR loci characteristics and primer sequences.

SSR ID	Primer ID	Primer Sequences (5′-3′)	SSR Motif (5′-3′)	Annealing T (°C)
GSSR-16 *	P080 P081	Fwd: ACTTTTGTTCCTGCATTACACAGT Rev: TGTGATGTTTGCAGGACATGG	(AT)_3_	59
GSSR-35 *	P082 P083	Fwd: TGCGCTCAGTCAATTGATTTACT Rev: TCAGACACCCCTTTGTTGTTTTC	(GA)_5_; (GA)_3_; (GA)_9_; (GA)_3_	61
GSSR-97	P084 P043	Fwd: GGCAAAGAAACAGATTTGGAGA Rev: CTGCCCTAGCATCAAAACAAAC	(GA)_4_; (AG)_11_	61
GSSR-138 *	P085 P086	Fwd: CCTCTTGCTGTTGTTGGTGA Rev: CCGTGGAAAGTCAGAATCATC	(GA)_3_	60
GSSR-154	P064 P065	Fwd: CTTATATGTGATGGCGTCGAAA Rev: GACTGCACCGCTCCTAACTC	(TC)_5_ (TC)_13_	59
BSSR-14	P08 9P067	Fwd: TACCCATAACTCAAGTTGGATAATTC Rev: AATGTCTAAACCCACTGATTTAAAAG	(TTA)_3_	58
BSSR-59	P070 P090	Fwd: GATGAACTTAGATCATGTGGGACT Rev: GTACAGCTGGTCAATCCGATG	(TA)_5_	58
BSSR-75	P091 P073	Fwd: ATGAAAGCAGGGATAAAAGTATCCAG Rev: AGAAGAAGGATTCAAGAAATGGCACA	(GCA)_3_; (TAT)_3_; (CTA)_4_	62
BSSR-91	P092 P075	Fwd: AGCTTCAACAGGGTCTTGAGTTC Rev: CTTGGCTCAACTTATGCAACTTCT	(TTG)_3_; (TTG)_4_	61

* primer specifically designed for fennel after sequencing.

**Table 3:** Genetic diversity as expressed in terms of allele size (bp), number of alleles (Na), effective number of alleles (Ne), observed (Ho) and expected (He) heterozygosity, inbreeding coefficient (F), polymorphic information content (PIC), null alleles frequency (NAF), and probability of identity (PID and PIDsib) of the 21 fennel accessions.

Locus	Range of Allele Size (bp)	Na	Ne	Ho	He	F	PIC	NAF	PID Unrelated	PIDsib
GSSR-16	293–305	3	2.12	0.050	0.529	0.906	0.406	0.8298	0.3439	0.5778
GSSR-35	249–253	3	1.17	0.150	0.145	0.035	0.136	0.0300	0.7428	0.8651
GSSR-97	256–268	5	3.65	0.650	0.726	0.105	0.656	0.0194	0.1372	0.4305
GSSR-138	390	1	1.00	0.000	0.000	-	0.000	ND	1.0000	1.0000
GSSR-154	300–370	12	6.49	0.400	0.846	0.527	0.810	0.3365	0.0456	0.3489
BSSR-14	222	1	1.00	0.000	0.000	-	0.000	ND	1.0000	1.0000
BSSR-59	222–230	2	1.63	0.300	0.385	0.221	0.305	0.1111	0.4609	0.6777
BSSR-75	174–242	2	1.11	0.000	0.097	1.000	0.090	0.7350	0.8235	0.9084
BSSR-91	174	1	1.00	0.000	0.000	-	0.000	ND	1.0000	1.0000
Mean	-	3.33	2.13	0.172	0.303	0.454	0.267	-	-	-
Total	-	30	-	-	-	-	-	-	6.06×10^−4^	4.62×10^−2^

**Table 4:** Varieties used in this research. Samples provided by Enza Zaden are named EZ coupled with a number and referred to a not declared F_1_.

Varieties	Company	Varieties	Company
Bianco Extra	Fratelli Ingegnoli	Wadenromen	FOUR - Blumen Group
Carmo	Fratelli Ingegnoli	EZ#1	Enza Zaden
Chiarino	Fratelli Ingegnoli	EZ#2	Enza Zaden
Cristal	Fratelli Ingegnoli	EZ#3	Enza Zaden
Mantovano	FOUR - Blumen Group	EZ#4	Enza Zaden
Montebianco	Fratelli Ingegnoli	EZ#5	Enza Zaden
Romanesco	Dom Sementi - SDD	EZ#6	Enza Zaden
Romanesco *sel.* Circeo	FOUR - Blumen Group	EZ#7	Enza Zaden
Selvatico	Fratelli Ingegnoli	EZ#8	Enza Zaden
Tauro	Fratelli Ingegnoli	EZ#9	Enza Zaden
